# Yeast-Derived Products: The Role of Hydrolyzed Yeast and Yeast Culture in Poultry Nutrition—A Review

**DOI:** 10.3390/ani12111426

**Published:** 2022-05-31

**Authors:** Vera Perricone, Silvia Sandrini, Nida Irshad, Giovanni Savoini, Marcello Comi, Alessandro Agazzi

**Affiliations:** 1 Department of Veterinary Medicine and Animal Sciences, University of Milan, Via dell’Università 6, 26900 Lodi, Italy; vera.perricone@unimi.it (V.P.); silvia.sandrini@unimi.it (S.S.); nida.irshad@unimi.it (N.I.); giovanni.savoini@unimi.it (G.S.); 2 Department of Human Science and Quality of Life Promition, Università Telematica San Raffaele, Via di Val Cannuta 247, 00166 Rome, Italy; comimarcello@gmail.com

**Keywords:** hydrolyzed yeast, yeast culture, poultry, performance, immune response, gut health

## Abstract

**Simple Summary:**

Yeast and yeast-derived products are largely employed in animal nutrition to support animals’ health and to improve their performance. Thanks to their components, including mannans, β-glucans, nucleotides, vitamins, and other compounds, yeasts have numerous beneficial effects. Among yeast-derived products, hydrolyzed yeasts and yeast cultures have received less attention, but, although the results are somewhat conflicting, in most of the cases, the available literature shows improved performance and health in poultry. Thus, the aim of this review is to provide an overview of hydrolyzed-yeast and yeast-culture employment in poultry nutrition, exploring their effects on the production performance, immune response, oxidative status, gut health, and nutrient digestibility. A brief description of the main yeast bioactive compounds is also provided.

**Abstract:**

Yeasts are single-cell eukaryotic microorganisms that are largely employed in animal nutrition for their beneficial effects, which are owed to their cellular components and bioactive compounds, among which are mannans, β-glucans, nucleotides, mannan oligosaccharides, and others. While the employment of live yeast cells as probiotics in poultry nutrition has already been largely reviewed, less information is available on yeast-derived products, such as hydrolyzed yeast (HY) and yeast culture (YC). The aim of this review is to provide the reader with an overview of the available body of literature on HY and YC and their effects on poultry. A brief description of the main components of the yeast cell that is considered to be responsible for the beneficial effects on animals’ health is also provided. HY and YC appear to have beneficial effects on the poultry growth and production performance, as well as on the immune response and gut health. Most of the beneficial effects of HY and YC have been attributed to their ability to modulate the gut microbiota, stimulating the growth of beneficial bacteria and reducing pathogen colonization. However, there are still many areas to be investigated to better understand and disentangle the effects and mechanisms of action of HY and YC.

## 1. Introduction

Poultry products (meat and eggs) are among the products of animal origin most widely eaten worldwide, and they represent excellent sources of protein and other nutrients. Their consumption has been steadily increasing over the last 50 years, and this is expected to further expand in the coming future [1]. Great improvements have been made in the production efficiency to meet the market demand by means of genetic selection; however, it is recognized that animals with high growth and production performances need to maintain an optimal health status in order to be able to fully express their genetic potential [2,3]. Among the strategies that have been adopted to sustain animals’ health, nutrition plays a key role. In this light, numerous feed additives and nutraceuticals have received significant attention due to their potential, and yeast is one of the most prominent [4,5].

Yeasts are single-cell eukaryotic microorganisms that are classified in the fungi kingdom [6]. There are about 500 different yeast species, but only a few of them are commercially exploited, with *Saccharomyces cerevisiae* being the predominant one. *S. cerevisiae*, which has QPS (Qualified Presumption of Safety) status according to the European Feed Safety Authority (EFSA), is largely used in the feed industry. Other yeasts that find application in animal production, although to a lesser extent, are *Kluyveromyces marxianus* (also referred to as whey yeast) and *Candida utilis* [5]. There are numerous forms of yeast and yeast-derivatives available on the market, including viable yeasts, administered for their probiotic activity, and fractionated yeasts (i.e., yeast-cell components, such as β-glucans and mannans), fed for their prebiotic activity. Besides them, yeasts are also available as specialty yeast products, such as selenium yeast and nutritional yeasts, which are used for their relatively high nutrient contents (proteins, amino acids, energy, and micronutrients). While pro- and prebiotic yeasts in the livestock and poultry sectors have been already largely reviewed [5,7,8,9], this review paper will focus on the employment in poultry production of two less-explored yeast products: hydrolyzed yeasts and yeast cell culture.

Hydrolyzed yeast is a relatively novel feed material, which can be obtained through different strategies, with the autolysis and hydrolysis processes being the most employed [10]. Autolysis is a degradation process that relies on the activation of the intracellular enzymes to solubilize the cell components within the cell, while enzymatic hydrolysis relies on different digestive enzymes to break the cell wall [11,12]. Therefore, hydrolyzed yeast consists of the total content of the yeast residue from the lysis process, and thus contains B-vitamins, amino acids, nucleotides, ß-glucan, and mannan oligosaccharides [13]. The choice of the yeast lysis process is strictly related to the content recovery; the enzymatic-hydrolysis method, as compared to other processes, such as autolysis, is significantly faster and has a higher recovery yield [10]. For the sake of this review, we refer to the generic term ‘hydrolyzed yeast’ (HY) when the lysis method was not specified by the authors, and to ‘enzymatically hydrolyzed yeast’ (EHY) and ‘autolyzed yeast’ (AY) when enzymatic hydrolysis or autolysis was performed, respectively.

Yeast culture (YC) is a unique micro-ecological product that is composed of yeast biomass (including some residual viable cells, dead cells, and yeast-cell-wall fragments), fermentation metabolites, and the residual growth medium [14]. YC contains a rich variety of biologically active substances, including proteins, small peptides, oligosaccharides, vitamins, minerals, enzymes, and numerous ‘unknown growth factors’, which can all exert beneficial nutritional and health effects on animals. YC is produced by inoculating live yeast cells in a specific culture medium, which are then allowed to ferment under defined conditions, upon which time the entire fermented medium is subsequently dried [5]. Therefore, the composition of the fermentation medium and the fermentation conditions influence the composition of the produced metabolites [15]. The most commonly employed YC in poultry production is produced by the fermentation of *S. cerevisiae*, which is also referred to as *Saccharomyces Cerevisiae* Fermentation Product (SCFP) [16]. Due to the predominant role played by the fermentation metabolites, YC and SCFP can be considered under the umbrella of postbiotics. Postbiotics is a relatively new concept that refers to factors (products or metabolic byproducts) secreted by live microorganisms or released after their lysis, with physiological benefits to the host [17].

This review aims to provide the reader with an overview of the available body of literature on hydrolyzed yeast and yeast culture, and their effects on poultry. To facilitate the understanding of the mechanisms of action, a brief description of the main components of the yeast cell that is considered responsible for the beneficial effects on animals’ health is also provided.

## 2. Yeast Bioactive Compounds

The cell wall of yeast is mainly composed of polysaccharides and proteins [18]. The former makes up nearly 75–85% of the cell wall, while the latter makes up the remaining 15–25%. Long-chain polysaccharides of the cell wall include water-soluble mannans, water-soluble and water-insoluble glucans, and chitin [19]; α-d-mannans and β-d-glucans represent the two main types of polysaccharides. Cell-wall polysaccharides are present in different proportions, depending on the yeast species; however, in *S. cerevisiae*, the amount of mannans and glucans is almost equal [20]. On the other hand, proteins form a covalent bond with mannans in the form of mannoproteins.

Mannan is the generic name for the polysaccharide moiety of glycoproteins [21], which is usually represented by a linear polymer of linked mannose residues [22]. Yeast mannan contains an α(1–6)-linked backbone and α(1–2)-linked and α(1–3)-linked branches [23]. The α-d-mannans have been shown to have antioxidant properties [24] and an inhibition ability against mycotoxins’ harmful action by interacting with their hazardous radical metabolites [25]. Many intestinal bacterial pathogens have mannose-specific lectins, which mediate adhesion and subsequent colonization and infection via the mannose-rich epithelial surface of the gut and intestines [26]. Korcová et al. [27] found that mannans stimulate macrophages in vivo via interaction with their transmembrane glycoprotein receptors, which recognize mannosylated compounds and facilitate their endocytosis, participating in the pathogen clearance [28]. Moreover, the interaction of mannans with the macrophage mannose receptor, and the subsequent stimulation of phagocytosis, actively induce the release of secretory products, such as IL-1, TNF, and reactive oxygen intermediates, which further boosts antigen clearance [29]. As a result, they can help to remove circulating atherogenic lipoproteins via engaging macrophages [30].

Glucans, as d-glucopyranosyl-based polysaccharides, are formed by the covalently bonded combination of (1→3)-β-d-glucan and (1→6)-β-d-glucan. In poultry, β-glucans have been shown to improve gut health and to positively affect the immune response through an anti-inflammatory effect [31]. β-glucans have been proven to boost white-blood-cell growth, which stimulates the immune system. The β-glucans are delivered to the small intestine and are then transmitted through the gut-associated lymphoid tissue’s Peyer’s patches and moved around the body [31]. β-glucans have been shown to change the intestinal profile of cytokines that activate the major histocompatibility complex and, subsequently, monocytes and macrophages [32]. β-glucans have also been found to improve the immunological response in hens by changing their cytokine profiles [33]. The increase in the immunological response includes the activation of T-helper cells, cytotoxic macrophages, and natural killer cells, as well as T-cell proliferation and differentiation [34]. The major signaling molecules involved in controlling this immunological network appear to be IL-1, IL-2, IFN-γ, and TNF-α [31]. The supplementation of β-glucan in the diet of poultry increased the number of goblet cells and increased the mucin-2 synthesis in goblet cells [35]. Goblet cells are cellular structures that keep the mucous barrier layers intact so that enteric infections can be removed effectively [36]. Moreover, one of the most important characteristics of yeast β-d-glucans is their ability to adsorb several mycotoxins [37,38,39], and, thus, their inclusion in animal feed may lead to the suppression of these compounds’ hazardous effects. Among the numerous beneficial effects associated with β-glucan administration, the most significant results have been found with β-glucan obtained from the yeast *Saccharomyces cerevisiae* [40]. Due to their reported properties, β-glucans may provide a new method for reducing antibiotic use in chicken diets [35].

Chitin (CT) is a linear polysaccharide that is made up of N-acetylglucosamine (N-acetyl D-glucosamine) and found in the yeast’s exoskeletons at a considerably lower concentration than mannans and glucans [5,41]. Contrary to other animal species, birds can digest chitin, either because of the endogenous chitinase enzyme [42] or because of commensal microorganisms in the gut or prey [43]. Due to their positive biological properties, such as biodegradability, biocompatibility, non-antigenicity, and nontoxicity, CT and chitosan have received increasing attention from researchers in the last years [44,45]. Hossain and Blair [43] observed that chitin has some nutritional value as a feed ingredient and could be fed to poultry with no adverse effects, although its ash content is quite high (260 g/kg), and the energy value of isolated chitin is 25 to 30% lower than that of the regularly used grains in chicken feeds. Chitin’s protein digestibility and amino acid content suggest, anyway, that it could also be useful as a protein and amino acid source for poultry [43].

Mannan oligosaccharides (MOS) are prebiotics and nutrient sources for certain microorganisms in the gastrointestinal tract, which can stimulate and promote the growth of beneficial bacteria in the gut [46]. In their review, Spring et al. [46] analyzed 733 published experiments regarding the effects of feeding MOS to companion animals, horses, rabbits, chickens, pigs, calves, and other aquaculture species, and found that MOS generally improved the body-weight gain and feed conversion, while lowering mortality. MOS binds to pathogens in the gastrointestinal tract, which limits their colonization, enhances the integrity of the intestinal mucosa, influences the immune-system activity, and is probably involved in antioxidant and antimutagenic defenses [47]. Dietary MOS was found to modify the intestinal expression of the crucial genes for the cell immunological response, including 2-microglobulin, lysozyme, lumican, apolipoprotein A-1, and fibronectin, which confirms the immunomodulating effect of MOS at the transcriptional level [48]. In vaccinated chickens, MOS at 1, 2, or 3 g/kg in the diet were also able to boost the humoral immune response and increase the antibody titer against avian influenza virus [49].

Mannoproteins (MPTs) are glycoproteins that contain 15–90% mannose by weight and are related to other cell-wall components, such as β-glucan and chitin [50]. MPTs have a molecular mass of 100 to 200 kDa, and they account for roughly 40% of the dry weight of the cell wall [51]. Immune modulators with immune-altering actions [52] and immunostimulatory-boosting dendritic cells and T-cell activity have been discovered in MPTs [53]. In necrotic enteritis-affected birds, a feed additive containing mannoprotein and β-glucan made up of pure yeast-cell-wall components was able to restore the body-weight increase while also providing unique cellular signal transduction in the gut of challenged broilers [54]. The positive responses to mannoprotein and β-glucan supplementation in poultry appear to be oriented toward the stimulation of cell growth while minimizing cell death and apoptosis, as well as innate inflammatory responses. Specifically, as a mechanism of action, mannoprotein and β-glucan supplementation seemed to induce compensatory signals to lessen the severity of the illness, rather than returning the tissue to a nonchallenged state [54].

Nucleotides are a class of bioactive molecules with low molecular weights and intracellular properties that play a vital role in animal physiological functions [55]. Gil [56] and Sauer et al. [57] regard dietary nucleotides to be key nutrients for modulating the immunological and gastrointestinal functions and optimizing the gut flora. There is growing evidence that supplementing nucleotides into monogastric animals’ diets can affect the intestinal morphology and function, the immune response, the intestinal microbiota composition, the liver function and morphology, and the growth performance; thus, knowing the nucleic acid content of yeast and yeast-containing products is critical [57,58]. Supplementing birds with yeast nucleotides can help to produce a faster and stronger antibody response to the IBV vaccine [59], enhancing intestinal growth and barrier-related gene expression, as well as the diversity and richness of the intestinal microbiota [59].

In addition to the bioactive molecules previously described, yeast cells are a source of additional elements, such as vitamins, minerals, and a well-balanced amino acid profile, together with other functional constituents, such as phenolic acids, flavonoids, carotenoids, and peptides [60,61,62,63].

## 3. Hydrolyzed and Autolyzed Yeast

### 3.1. Effects on Growth Performance and Egg Production

The use of HY in poultry is generally able to improve the growth performance in terms of the body-weight gain (BWG) and a better feed conversion ratio (FCR), with no evidence on feed intake (FI) and mortality [64,65]. The HY efficacy, anyway, seems variable, accounting for the different rearing stages of poultry; improved performance was obtained at the beginning of the rearing cycle [66,67] or during the grower period only [68,69]. Besides the rearing phase, the HY efficacy seems to be dependent on the amount included in the feed. Recent studies have evidenced it as graded inclusion levels, and, specifically, higher inclusion rates led to more positive results on the performance [64,65]. Interestingly, beneficial effects of HY supplementation (i.e., improved BWG and FCR) have also been reported in the progeny of broiler breeder hens fed 5 g/kg of HY [13].

Higher growth performance was observed when EHY or AY were administered to broiler chickens [66,70,71,72] and quails [73]. Overall increased BWG and an improved FCR in broilers supplemented with 1.5 g/kg of feed AY were found by Ullah et al. [71] and Ahiwe et al. [66].

Beneficial effects of HY and AY administration have also been reported on the production performance of breeder and laying hens. Specifically, the dietary inclusion of AY in laying hens’ diets improved egg production and weight [74,75,76]. On the other hand, Araujo et al. [13] found that HY inclusion in the diet of breeder hens led to increased egg production (+2.14%), fertility (+1.77%), and hatchability for incubated and fertile eggs (+4.79% and +2.56%, respectively).

Generally, the positive effects of HY, EHY, or AY on broiler and breeder performance may be associated with their pre- and probiotic activities on the intestinal microbes, which result in a healthier gut and better feed utilization. The improved nutrient utilization, as well as nutrient deposition in the eggs used for embryo development, result in increased BWG and an improved FCR in poultry [66,70] and their progeny [13], respectively. The beneficial effects observed on the growth performance could be explained by the modulation of the gut microbiota exerted by yeast components (i.e., MOS and β-glucans), which results in an increased production of short-chain fatty acids, which serve as an energy source for the enterocytes [68].

### 3.2. Effects on Slaughter Yield and Product Quality

Most of the available literature reports on the beneficial effects of HY on the growth performance of broiler chickens can be directly reflected in their performance at slaughtering. Different studies have evidenced that HY, AY, and EHY improved the slaughter performance in broiler chickens or breeder layers’ progeny when administrated in the diet at an inclusion level ranging from 0.5 to 5 g/kg [13,66,70,72]. The most affected parameters were the carcass yield and the absolute and relative breast and drumstick weights. Beneficial effects of AY on the slaughter performance have been confirmed in quails, where the slaughter weight and the hot and cold carcass weights were found to be increased, although with some differences according to gender [73]. At the same time, AY was also found to determine a decrease in the relative abdominal-fat weight in broilers [67]. Only one study, by Ullah et al. [71], failed to observe a significant contribution of AY to the slaughter performance

In terms of the effects on the egg quality, Yalçın et al. [74,75] observed that the dietary inclusion of AY to laying hens’ diets decreased the egg-yolk-cholesterol content [74,75] and affected the fatty acid composition, increasing the total saturated fatty acids and the ratio of saturated/unsaturated fatty acids while decreasing the total monounsaturated fatty acids [74].

The mechanism of action behind the ameliorated performance at slaughtering and the product quality could be reconducted to the role of HY components in ameliorating the gut environment, which finally results in better feed utilization and nutrient delivery towards the production parameters [13,74,75].

### 3.3. Effects on the Immune Response and Oxidative Status

HY has been associated with the modulation of the immune response in poultry, acting on both the cell-mediated and humoral immunity (Table 1) [67,68].

Bortoluzzi et al. [68] report that the administration of 2 and 4 g/kg of AY in the diet of broiler chickens could improve the local immunity, as is suggested by the trend toward a lower number of B lymphocytes, the reduced number of helper T cells, and the decreased number of activated T-cytotoxic lymphocytes in the bloodstream. The authors hypothesized that the trend of a lower number of these cells in the blood could be justified by their movement to the lamina propria of the intestine, which thus improves the local immunity. In the same study, the effect of AY on the immune gene expression in ileal tissue was also investigated. Although yeast products have been reported to stimulate the innate immune response of animals by modulating the expression of toll-like receptors (TLR) and cytokines [77], Bortoluzzi et al. [68] only evidenced a significant upregulation of IL-1β on d 21 of life with an AY supplementation of 4 g/kg in the feed. The authors suggest that the upregulation of IL-1β may be responsible for the intestinal recruitment of lymphocytes, which is in agreement with the lower T-cytotoxic cells in the blood previously mentioned. According to the findings of Bortoluzzi et al. [68], and also Ahiwe et al. [70], the AY supplementation in broiler chickens resulted in the modulation of or decrease in the white blood cells (WBCs) and the lymphocyte and monocyte counts when the animals were challenged with LPS. To the contrary, Attia et al. [78] found a significant increase in white blood cells, lymphocytes, and heterophil numbers when broilers were fed HY.

Different authors have investigated the effect of HY on the humoral immune response, and mainly by means of vaccinations. Most of the works show that HY administration (1 g/kg or 2 g/kg) in the diet of broiler chickens resulted in higher antibody titers against Newcastle disease (NCD), avian influenza (AIV), and infection bursa disease (IBDV) [64,69,78], although a lack of efficacy was eventually found on NCD titers and the immunoglobulin A levels [64,70].

By using sheep-red-blood-cell (SRBC) injection, Yalçın et al. [67,74,75] increased the antibody production in broiler chickens [67] and laying hens [74,75], who received 2 g/kg of AY in the diet.

To assess the effects of HY on the immune response, some authors evaluated the weight of immune organs, such as the spleen and the bursa of Fabricius, reporting contrasting results. Attia et al. [78] report that the administration of HY resulted in increased thymus and bursa relative weights, and a larger follicle diameter of the bursa. On the other hand, Ahiwe et al. [79] report a reduced spleen weight in broilers fed hydrolyzed-yeast-cell-wall extract and challenged either with *Eimeria* or *C. Perfrigens*. Nevertheless, Ahiwe et al. [70] found that the administration of 2 g/kg of feed of AY in the diet of broiler chickens challenged with *Salmonella* lipopolysaccharides resulted in no significant differences in the relative weights of the spleen and bursa.

Besides modulating the immune response, HY could also have the potential to modulate the oxidative status of animals. Although only a little information is available in this regard, HY administration to broiler chickens was found to modulate the serum concentrations of SOD, GSH, GPX, and TAC [78].

### 3.4. Effects on Gut Health

Among the proposed mechanisms of action residing behind the beneficial effects of HY, the modulation of the gut health, and mainly the modulation of the gut microbiota, could play a pivotal role. HY are reported to enhance the gut microbial profile, although there is a lack of studies that have investigated, in depth (e.g., by means of next-generation-sequencing (NGS) technologies), the effects of HY on the intestinal microbial profile of poultry. To date, and to the best of the authors’ knowledge, all the studies that have evaluated the effect of HY on gut ecology have relied on cultivation-dependent approaches (Table 2).

Different authors have investigated the effect of HY on the *Lactobacillus* count, reporting a linear increase in the excreta and ileal digesta of broilers receiving 1 and 2 g/kg of feed HY [65,69], as well as in laying hens receiving 0.5, 1, 5, 10, or 30 g/kg of HY [90].

Other authors have investigated the potential of HY to control pathogens, reporting contrasting results. Park et al. [90] observed a linear reduction in *E. coli* in the excreta of laying hens fed different levels of HY, which is in line with the results observed in broiler chickens receiving HY [65] or AY [67]. Similar results are also reported by Muthusami et al. [69], who observed lower counts of *E. coli* and *Salmonella spp.* in the digesta of HY-fed broilers. However, the same authors also observed the opposite trend at the mucosal level, reporting higher counts of *E.coli* and *Salmonella* in the small-intestine mucosa. The authors suggest that this finding could be explained by the increased mucin secretion from the goblet cells under the influence of HY. Mucin and its components can competitively attach to Type-I fimbriae or Gram-negative bacteria, which finally results in increased counts of bacteria at the mucosal level and a reduced number of free bacteria in the lumen.

As a consequence of gut-microbiota modulation, HY may have a role in intestinal pH regulation, as observed by Yalçın et al. [67], where 2, 3, and 4 g/kg of feed AY resulted in a reduction in the pH values of the jejunal and ileal digesta.

Besides modulating the gut microbiota, HY and AY administration could improve gut health by modulating the intestinal morphology. Muthusami et al. [69] first, and Sampath et al. [65] recently, report an increased villus height in the jejunum, an improved villus width in the ileum, and an increased number of goblet cells in the duodenum, jejunum, and ileum of HY-supplemented broilers. However, the crypt depth and the thickness of the muscularis mucosae layer were not affected by dietary treatment. Nevertheless, only a little information is available at the transcriptional level; Bortoluzzi et al. [68] report that 2 and 4 g/kg of AY inclusion in the diet of broiler chickens did not affect the Claudin-1 and MUC-2 gene expression.

### 3.5. Effects on Nutrient Digestibility and Utilization

Hydrolyzed and autolyzed yeast have the potential to improve nutrient digestion and absorption by ameliorating animal-gut health (Table 3). Moreover, a few studies that were conducted in the past on the nutrient digestibility and utilization in poultry receiving HY show that a general increase was observed in different nutrients. Improved ileal dry matter, nitrogen, protein, ashes, and energy digestibility were found in most of the cases [65,66,72], at inclusion rates in the feed ranging from 1 to 2 g/kg of HY or EHY.

The observed increased nutrient digestibility and utilization in poultry can be attributed to the HY components, such as β-glucan and mannan oligosaccharides, which can act as substrates for beneficial microbe proliferation or for explicating a trophic activity on the intestinal mucosa that results in a higher villus height [101]. Besides the cited effects, the other suggested mechanisms of action of HY seem to rely on increased pancreatic-protein-enzyme activity (higher pancreatic-tissue-protein content) and the higher activity of trypsin and chymotrypsin, which lead to improved ileal protein digestibility [66]. Moreover, it has been noticed that MOS, itself, could be a good source of digestive enzymes, such as amylase, protease, and lipase [102].

References on the supplementation of HY in laying hens are very scarce but, similar to what was observed in broilers, a linear increase in the dry matter and nitrogen digestibility were found when administering different HY amounts in the feed (0.5, 1, 5, 10, or 30 g/kg). However, while in broiler chickens, HY and EHY were found to improve the energy digestibility, the result was not confirmed in laying hens, where the energy digestibility showed no significant differences between the control animals and the animals receiving HY. [90].

## 4. Yeast Culture

### 4.1. Effects on Growth Performance and Egg Production

YC administration has been associated with the improved growth performance of broiler chickens, with the main significant results observed on BW and BWG, although with variable efficacy, accounting for the different rearing stages of poultry. In particular, some authors report improved BWG in the first phase of the production cycle, when the YC was administered either alone [103] or in combination with phytase [84]. In the last case, the authors suggest the hypothesis that the metabolic products (i.e., postbiotics) of YC could act synergistically with phytase, leading to increased nutrient utilization and decreased phytate content, which finally results in an improved growth performance. On the contrary, other authors observed a significant effect of varying levels of YC on the BW and the average daily gain (ADG) in the grower phase and over the entire experimental period (d 1 to 42 of age), but not at earlier stages [81,82,104]. While the main effects have been observed as improved BW and BWG, most of the studies in the literature report that YC supplementation in broilers was not effective on the FI [85,93,103,105,106,107], with few exceptions [82,98]. Consequently, in most of the studies, YC did not affect the FCR [82,85,103,106,107], although Gao et al. [81] observed an improved feed-to-gain ratio in the grower phase and over the entire experimental period when broiler chickens received 2.5 g/kg of YC.

As the beneficial effects of YC on the growth performance have been suggested to be linked to the reduction in stress and the modulation of the immune response, some researchers applied challenging conditions, confirming the beneficial effects of YC supplementation on BW and BWG after Salmonella infection [108], live coccidiosis vaccine [95], or exposure to a diet containing aflatoxins [109], which is contrary to a few others [110].

While most of the studies were conducted on broiler chickens, few studies have investigated the effects of YC in other poultry species, and, even in this case, the most relevant results are related to an increased BW rather than FI or FCR. In laying hens and layer pullets, the supplementation of varying levels of YC resulted in increased BW and BWG under both challenging [86,87,111] and nonchallenging conditions [112]. Similarly, YC supplementation (either in the feed or in the water) improved the BW of turkey hens subjected to different types of stressors related to environmental conditions and management practices [113]. Nevertheless, other authors report no significant effect of YC supplementation on the growth performance of laying hens and layer pullets [114,115,116], or of turkeys [117,118].

The potential of YC administration has also been evaluated in laying hens to assess the effects on egg production. The results retrieved in the literature are, however, quite contrasting. Following the administration of levels of YC ranging from 0.7 to 4 g/kg, several authors report an increased laying rate [86,87,88,119] and egg weight [112,115]. However, other authors report no significant effect of YC on egg production or egg weight, even when the tested product was the same [114,116]. In some cases, differences were pointed out in the weights of individual egg components. For quails supplemented with 2 and 4 g/kg of YC, Cepuliene et al. [119] recorded that the egg white and egg yolk were increased and decreased, respectively. The opposite trend was observed by Martinez et al. [114], where the YC in the diet of laying hens reduced the percentage of the albumen yield and increased the percentage of the yolk yield.

The positive effects of YC have been attributed to, among other suggested mechanisms, its pro- and prebiotic effects. Thus, the supplementation of YC could stimulate the growth of beneficial bacteria and inhibit pathogens, maintaining an optimal microbial community and improving feed digestion as a result [83,100]. Another hypothesis for YC efficacy is a reduced animal susceptibility to stress, which consequently attenuates gut-barrier dysfunction [84,87]. However, the discrepancies in the observed results might be attributable to the composition of the product derived from, among other things, the production process. Sun et al. [91] investigated the effect of YCs obtained by different fermentation times (12, 24, 36, 48, or 60 h), reporting improved performances in broilers fed YC fermented for 12 and 24 h, whereas poor performances were associated with YC fermented for 48 and 60 h. As highlighted by the metabolomic analysis, YCs produced with different fermentation times contain different metabolites, which thus results in different effects.

### 4.2. Effects on Slaughter Yield and Product Quality

As previously stated, YC has the potential to improve the animal performance, which could be subsequently reflected in meat production (i.e., in the carcass yield and the yields of commercial cuts). However, in most of the studies, the administration of YC showed no effect on the carcass yields in broilers [103,104,120] and turkeys [117,121]. Only few studies report an improved slaughter performance, where better results were achieved when the YC was administered at high inclusion rates in the feed. Aristides et al. [122] conducted a study to evaluate the effect of YC at 0.25, 0.75, 1.5 g/kg of feed in broiler chickens and found an increased drumstick yield only in the group receiving the highest YC concentration. The effectiveness of high YC supplementation (1.5 g/kg feed or 2× and 3× the recommended inclusion rate, respectively) was previously confirmed by Fathi et al. [104] and Firmann et al. [121] on broiler and turkey-breast yields.

Thanks to its bioactive components, among which are some compounds with antioxidant activity, YC might affect the meat quality. However, few studies have investigated the impact of YC on the meat quality, and they report only minor effects. Artistides et al. [122] observed a reduction in the muscle pH and lipid oxidation (TBARS) when YC was included in the diet at inclusion rates of 1.5 and 0.75 g/kg, respectively. On the contrary, other studies report no effect of YC on the pH values of breast muscles [105], nor on the muscle-color parameters (brightness and intensity of red and yellow), the water-holding capacity, the cooking loss, or the shear force of broiler meat [105,122].

As a result of its composition, and especially of the presence of MOS and β-glucans, YC could modulate some of the qualitative traits of eggs. Yalcin et al. [112] report a decreased cholesterol content in egg yolk following the administration of 2 g/kg of YC in the diet of laying hens, while Oszoy et al. [115] observed the modulation of the yolk fatty acid composition, and especially of the C18:2 n6 content.

The observed effects of YC on the performance at slaughtering and the product quality have been attributed to improvements in either nutrient utilization, immune response, or gut health [87,121,122]; however, the discrepancies in the results could be ascribed to numerous factors, such as age, breed, diet composition, environmental conditions, and the duration of the experimental trial.

### 4.3. Effects on the Immune Response and Oxidative Status

YC and its active components have been described in multiple studies as modulators of the immune response, acting on both the innate and adaptive immune responses (Table 1). Yeast components and fermentation metabolites may help to balance the immune and stress response, while, at the same time, improve the antioxidant status [83,123].

YC administration has been shown to be effective in modulating the innate immune response of broiler chickens. Recently, Chou et al. [80] evaluated the effect of YC on the immune response of broilers challenged with a live attenuated Newcastle-disease-virus (NDV) vaccine. They report the increased gene expression of pattern-recognition receptors (PRRs), including TLR-2, in the spleen of broilers receiving 1.25 g/kg of YC in the diet. Since the ligation of TLR-2 with ligands could trigger different immunological events, among which is a peripheral tolerogenic response, the authors suggest the role of YC in maintaining the balance of the immune response and in preventing innate-immunity overreaction via the regulation of PRRs. Furthermore, YC-supplemented broilers showed a higher baseline expression of interferons (INFs), which participate in the maturation of Th1 cells, as well as in leukocyte attraction and the regulation of B-cell immunoglobulin production [124,125]. Thus, it has been hypothesized that YC can keep the innate immune system of supplemented animals in a standby mode, where it is prepared and ready to respond to immune stimuli faster than in control animals [80]. Additionally, the same authors also report increased serum lysozyme activity. Lysozyme is mainly secreted by phagocytes and it is a nonspecific immune effector; thus, its increase in treated birds suggests that more phagocytes were activated, enhancing the nonspecific immune response. Similar results were observed in breeder layers [83].

Besides modulating the innate immune response, YC might influence the adaptive immune response as well. It has been indicated that YC supplementation could improve the ability of broilers to establish a protective immunity via antigen-specific T-cell expansion, which further facilitates the proliferation and differentiation of CD4+ Th and CD8+ cytotoxic T cells [80]. The results obtained by Chou et al. [80] confirm what was already reported previously by Gao et al. [82], who observed increased counts of CD3+, CD4+, and CD8+ T-lymphocytes in the spleen and the blood of broiler chickens receiving 2.5 and 5 g/kg of YC in the diet. At the same time, YC can influence the humoral branch of the adaptive immune response. When broiler chickens were subjected to NDV vaccination [81] or *Eimeria tenella* infection [82], the circulatory IgM concentrations were found to be higher compared to the control animals. Similarly, also at the mucosal level, YC administration has been proven to be effective in modulating the immune response, as evidenced by the increased SIgA concentrations in the cecal tonsils [81] and jejunal mucosa [14] of broiler chickens. Nevertheless, some studies evidence no changes in the immunoglobulin titers following the administration of different levels of YC [83,100]; however, these results could be related to the lack of immune stimulation (e.g., by means of immunological challenge).

In compliance with the modulation of the immune response, YC administration has also been associated with a modulation of the inflammatory status, as highlighted by the reduced gene expression of IL-1β, NF-kB, iNOS, and INF-γ in broilers’ PBMCs following the administration of *S. cerevisiae*-fermented products [84,85]. However, in other recent studies, YC failed to modulate the inflammatory status and cytokine levels [126,127].

Besides modulating the immune and inflammatory response, YC could also exert antioxidant activity [128]. It has been reported that YC was effective in reducing the levels of plasma malondialdehyde (MDA) in Nicobari chickens [86,87] and PD 3 chicken lines [88,89] subjected to heat stress, which is normally associated with reactive-oxygen-species (ROS) production, and, therefore, with oxidative damage. Moreover, while the MDA levels were not different in aged breeder layers receiving 2.0 g/kg of YC in the diet, the total antioxidant capacity (TAOC) was found to be significantly improved compared to the control animals [83]. Besides these outlined positive effects, the available literature also reports a lack of efficacy on the oxidative status of the animals, as stated by Nelson et al. [126], where YC supplementation, either in the water or in the feed of broiler chickens, did not affect any indicators of oxidative stress.

Although a preeminent part of the published papers found positive results on the immune response or oxidative damage after YC administration in poultry, the mechanisms of action underlying these effects are not fully elucidated yet. One of the main roles in the modulation of the immune response is played by β-glucans, which have a well-documented effect on immune-cell activity [129]. Additionally, mannan oligosaccharides could also contribute by preventing pathogen colonization and supporting the development of the intestinal barrier [48,130].

### 4.4. Effects on Gut Health

YC is characterized by a rich variety of bioactive components, which are believed to play a pivotal role in the sustainment of intestinal health, modulating the gut microbiota, and sustaining the integrity of the gut barrier.

Lately, the intestinal microbiota has elicited increasing interest, and the advent of NGS technologies, among which are 16S rRNA, have paved the way to a completely new research frontier to unravel how host–microbiome interactions affect animal health. In this light, an increasing number of studies have evaluated the effect of YC on gut microbiota [83,91,126] (Table 2). Microbial-diversity analysis (alpha and beta-diversity) is an essential step in understanding how the tested product impacts the microbiota. The stability of the microbiota is directly related to age [131], and the sooner the microbiota reaches a stable diversity, the sooner the animals are more protected from pathogen invasion [132] while maximizing the metabolic fermentation and nutrient absorption [133]. The studies in the literature report a significant effect of YC administration on microbial diversity, indicating that YC might increase diversity and allow the microbiota to stabilize at an earlier age, compared to animals receiving the basal diet only [94,134]. Experiments conducted in vitro, where YC was incubated with the feed and the ceca content of broiler chickens [94,135] or turkeys [94], resulted in increased alpha and beta diversity, which stabilized over time. Changes in the gut microbial diversity due to YC administration in the diet were also confirmed by in vivo studies on aged breeder layers [83] and broilers [91]. Nevertheless, other studies report no effects [126].

Besides increasing the microbial diversity, YC has been proven effective in modulating the gut microbial composition, stimulating the growth of beneficial bacteria. Among the beneficial bacteria, there are several species of Bacteroides, which metabolize polysaccharides and oligosaccharides, providing nutrition to the host [136]. The phylum Bacteroidetes was found to be improved in the ileum of breeder layers [83] and the cecum of broiler chickens [91] following the administration of YC in the diet. Firmicutes is another phylum that is associated with beneficial effects due to its role in producing butyrate [137]. However, while Sun et al. [91] observed an increase in its relative abundance in the cecum of broilers receiving YC, an opposite trend was reported in the ileum of breeder layers [83]. YC administration has also been associated with a positive modulation of *Lactobacilli*. Liu et al. [83] report that the relative abundance of *Lactobacilli* in the ileal content increased from 14.30 to 49.83% when 2.0 g/kg of YC was administered to breeder layers, which is a result that was further confirmed by LEfSe analysis. Similar findings were also observed in the cecum of broiler chickens [91] and in an in vitro poultry-cecal-culture model [92]. Nevertheless, other authors observed no significant effect of YC on *Lactobacilli*, either with culture-dependent [85,105] or culture-independent [135] methods. Finally, other beneficial bacteria that have been found to be positively affected by YC administration include *Bifidobacterium* [93], *Ruminococcaceae* [93,94], and *Faecalibacterium* [91].

Several authors have focused on the ability of YC to control pathogens. Numerous studies prove the ability of YC to reduce the *Salmonella* prevalence in the ceca content of broilers [95,96], layer pullets [97], and turkeys [94], as well as Salmonella survival [96,135] and shedding in the faeces [108]. Interestingly, it has been pointed out in in vitro studies that YC is effective at controlling *Salmonella* only when gut microbiota are present [96,135], while, when YC was incubated alone with the pathogen, it showed no effect. Similar results have been reported for *Campylobacter jejuni*, where the inclusion of 10 g/kg of YC in an in vitro poultry-cecal-culture model resulted in a significant reduction in *C. jejuni* [92]. Nevertheless, other studies report no effect of YC administration on *Salmonella* [105,106], *E. coli* [105], or *C. perfringens* [84].

YC administration in the poultry diet has also been associated with improved intestinal-barrier function and morphology. YC has been found effective at enhancing the gene expression of tight junctions (Occludin, Claudin-1, and Zonula Occludens-1) in aged laying hens [100] and broilers [84]. Such a result has been attributed to the modulation of the gut microbiome, and specifically to an increased abundance of *Lactobacillus* and decreases in *E. coli* and *Salmonella* [100], as well as to the microbial products, such as SCFA [84]. Along with improving the gut-barrier function, YC administration has been proven to be effective in supporting the gut morphology and development, mainly increasing the villi height and V:C ratio in different segments of the intestine [84,85,98]. This effect has been confirmed in poultry species under heat-stress conditions. High ambient temperature induces the production of ROS, which, in turn, can increase the intestinal permeability and cause damage to the membranes [138]. YC administration was able to alleviate the intestinal damage induced by heat stress in Pekin ducks [127] and broiler chickens [126], as highlighted by the increased villi height and V:C ratio, and the reduced crypt depth. The ability of YC to alleviate intestinal damage induced by heat stress was also confirmed by the reduced severity of villi necrosis in the jejuna of a PD 3 chicken line [88] and Nicobari chickens [86]. Nevertheless, some authors observed no significant effect of YC on the intestinal morphology of broiler chickens [103] and turkeys [121]. The discrepancies in the observed results could be attributable to the inclusion rate of the tested product, as advised by Gao et al. [81], who observed varying results in the guts of broiler chickens fed 2.5, 5, and 7.5 g/kg of YC, which suggests a dose-dependent effect. At the same time, the administration route could play a role as well, as indicated in the study of Nelson et al. [126], where only the administration of YC via the drinking water was effective at increasing the villus height in the ileum of animals exposed to heat stress.

The beneficial effect of YC on gut health is therefore clearly related to the modulation of the gut microbiota, which can, directly and indirectly, regulate the immune response of the animal, protect against epithelial damage and pathogen invasion, and increase the nutrient availability and digestibility [134]. Such effects could be attributed to the pro- and prebiotic effects of YC, to be reconducted to the action of some residual viable cells and the presence of α-mannans and β-glucans, respectively [139].

### 4.5. Effects on Nutrient Digestibility and Utilization

Thus far, only a limited number of studies have evaluated the effect of YC administration on nutrient digestibility and utilization in poultry, and they report minor effects (Table 3). Few studies indicate improvements in the crude-fat (EE) digestibility in aged breeder layers and broiler chickens [83,93], while only one study reports an improved apparent digestibility of crude protein (CP) in broilers [98]. However, in most cases, the EE, CP, and energy digestibility were not affected by YC supplementation [81,83,99,105]. While only minor effects have been reported for CP, EE, and energy, YC seems to play a role in improving the digestibility of Ca and *P*. Akhavan-Salamat et al. [99] report increased Ca and P ileal apparent digestibility when YC was administered to broiler chickens receiving a diet with inadequate levels of P. This finding is of particular importance, as the improved utilization of the minerals present in the diet could contribute to reducing their excretion in the manure, with special regard to P. It has been speculated that such results could be partially related to the phytase activity of YC [81,93].

Although nutrient digestibility seems to be scarcely affected by YC administration, Zhang et al. [100] report the increased activity of digestive enzymes (α-amylase and chymotrypsin) in the duodenum of laying hens receiving 3.0 g/kg of YC in the diet. These enzymes play a pivotal role in nutrient digestion, and they have been described as valuable parameters for feed utilization. Their improved activity could indicate the enhanced digestibility of protein and starch components, as supported by the improved feed conversion observed in the study [100]. Similar results are reported by Wang et al. [14], where the administration of a YC plus enzymatically hydrolyzed yeast cell wall in the diet of broiler chickens resulted in the improved activity of jejunal sucrase and maltase (i.e., two disaccharidase enzymes that play a crucial role in the digestion and absorption of carbohydrates). It is possible to reconduct the increased enzyme activity to the role that YC plays in supporting gut development, as YC sustains villi growth, and enzymes are secreted from the tip of the same villi [81].

## 5. Conclusions and Future Perspectives

Yeast and yeast-derived products have been largely investigated over the last decades, and they have found wide employment in the poultry industry. In this review, we summarized the main effects of HY and YC.

HY and YC appear to have beneficial effects on the poultry growth performance, particularly improving BW and BWG, with no or only minor effects on the FI, FCR, and mortality. In both cases, however, the results were found to be variable, accounting for the phase of the production cycle. Similarly, HY and YC showed beneficial effects on the production performance, improving the egg production and quality traits, although with some differences. On the other hand, only HY seems to be effective at improving the slaughter performance, while YC had mainly no effect.

Due to their bioactive components, and mainly to the presence of mannans and β-glucans, both HY and YC can modulate the immune response, acting on both innate and adaptive immunity (e.g., by modulating the lymphocyte populations and stimulating antibody production). The numerous bioactive compounds contained in the yeast cell also have antioxidant properties; YC has been found to reduce the oxidative damage induced by ROS molecules that are produced under heat-stress conditions. However, very little information is available on the effect of HY on the oxidative status of animals, which thus requires further investigation.

Most of the beneficial effects of HY and YC have been attributed to their ability to modulate the gut microbiota. Both products have been shown to stimulate the growth of beneficial bacteria, such as Lactobacilli, and to reduce the colonization of pathogens. However, while for YC, the composition of the gut microbiota has been evaluated by means of NGS technologies, this kind of information is still lacking for HY.

At the same time, only a few studies investigate the effects of HY and YC on the nutrient digestibility, and they show minor effects. However, YC administration was found to improve the Ca and P digestibility of broiler chickens receiving a basal diet with inadequate levels of P. This finding is of a particular interest and requires further investigation, as the improved utilization of the minerals present in the diet could contribute to reducing their excretion in the manure, thus mitigating the environmental impact of poultry farming.

Although HY and YC administration appears to have beneficial effects on poultry performance and health, the results reported in the literature are, however, quite contrasting. The observed discrepancies could be attributable to numerous factors, including age, breed, diet composition, environmental conditions, and the duration of the experimental trial. Furthermore, another important aspect to be considered is the production process, which has a strong influence on the final compositions of both HY and YC. For HY, the lysis process is strictly correlated with the content recovery, but in most of the studies reported in the literature, very little information is provided on the production (e.g., which kind of lysis process was employed). Similarly, for YC, the fermentation media and the fermentation conditions (e.g., pH, temperature, fermentation time) influence the final composition of the product, and especially the produced metabolites. In most of the studies, the YCs tested were commercial products, and no information was provided on their production or metabolite compositions.

In conclusion, HY and YC appear to be effective at sustaining poultry health and production. However, further studies are required to better understand their effects and to optimize their employment. A systems-biology approach should be applied to better understand the mechanisms of action of both products.

## Figures and Tables

**Table 1:** Main effects of hydrolyzed-yeast (HY), autolyzed-yeast (AY), and yeast-culture (YC) administration on the immune response of poultry.

Yeast Product	Animal Type	Challenge	Effect	Reference
AY	Broiler	Live vaccine against coccidiosis	↓ TLR-4 and ↑ IL-1β expression	[68]
AY	Broiler	Salmonella lipopolysaccharide	↓ spleen and ↑ bursa weight ↑ WBC count, albumin, and IgG	[79]
HY	Broiler	NDV, IBV, avian-influenza-vaccination protocol	↑ antibody titer	[64,69]
			↑ serum antioxidant enzyme, IgY, IgM, and IgA	[78]
AY	Broiler laying hens	SRBCs	↑ antibody titers to SRBCs	[67] [74,75]
YC	Broiler	NDV vaccine	↑ TLR-2 expression	[80]
			↑ IgM and SIgA	[81]
YC	Broiler	*Eimeria tenella*	↑ CD3+, CD4+, CD8+ T-lymphocytes, serum lysozyme, and IgM	[82]
YC	Breeder layers		↑ CD3+, CD4+, and CD8+ T-lymphocytes	[80]
			↑ TAOC and lysozyme	[83]
YC + HY	Broiler		↑ IgG and SIgA	[14]
SCFP ^1^	Broilers’ PBMCs		↓ IL-1β, NF-kB, iNOS, and INF-γ expression	[84,85]
YC	Nicobari chickens and PD 3 chicken lines	Heat stress	↓ plasma MDA	[86,87,88,89]

^1^*Saccharomyces cerevisiae* fermentation product.

**Table 2:** Main effects of hydrolyzed-yeast (HY), autolyzed-yeast (AY), and yeast-culture (YC) administration on gut microbiota of poultry.

Product	Model	Effect	Method	Reference
AY	Broiler	↑ *Enterococcus* and *↓ Lactobacillus*	Bacterial cultures	[68]
		↓ *E. coli*	Bacterial cultures	[67,68]
HY	Broiler	↑ *Lactobacillus* and *↓ E. coli*	Bacterial cultures	[65]
	laying hens			[90]
YC	Breeder layers	↓ Firmicutes	16s rRNA	[83]
		↑ Bacteroidetes and Firmicutes: Bacteroidetes		
YC	Broiler	↑ Bacteroidetes and Firmicutes	16s rRNA	[91]
YC	Breeder layers;	↑ Lactobacilli	16s rRNA	[83]
	broiler			[91,92]
YC	Broiler	↑ Bifidobacterium	16s rDNA/16s rRNA	[93]
		↑ Ruminococcaceae		[93,94]
		↑ Faecalibacterium		[83]
YC	Broiler	↓ *Salmonella*	Bacterial cultures	[95,96]
	layer pullets and			[97]
	turkeys			[94]
YC	Broiler	*↓ Campylobacter jejuni*	16s rRNA	[92]

**Table 3:** Main effects of hydrolyzed-yeast (HY), autolyzed-yeast (AY), and yeast-culture (YC) administration on nutrient digestibility of poultry.

Product	Model	Effect	Reference
AY	Broiler	↑ tissue protein content, pancreatic enzyme activities, and protein digestibility	[66]
HY	Broiler	↑ DM and N digestibility	[65]
	laying hens		[90]
EHY ^1^	Broiler	↑ ashes and energy digestibility	[72]
YC	Aged breeder layers and	↑ fat digestibility	[83]
	broiler		[93]
YC	Broiler	↑ CP digestibility	[98]
YC	Broiler	↑ Ca and P digestibility	[81,93,99]
YC	Laying hens	↑ α-amylase and chymotrypsin	[100]
YC + EHY	Broiler	↑ sucrase and maltase	[14]

^1^ enzymatically hydrolyzed yeast.

## Data Availability

Not applicable.

## References

[1] OECD/FAO (2021). OECD-FAO Agricultural Outlook 2021–2030.

[2] Kim W.H., Lillehoj H.S. (2019). Immunity, immunomodulation, and antibiotic alternatives to maximize the genetic potential of poultry for growth and disease response. Anim. Feed Sci. Technol..

[3] Flock D.K., Laughlin K.F., Bentley J. (2005). Minimizing losses in poultry breeding and production: How breeding companies contribute to poultry welfare. World Poult. Sci. J..

[4] Pandey A.K., Kumar P., Saxena M.J., Gupta R., Srivastava A., Lall R. (2019). Feed additives in animal health. Nutraceuticals in Veterinary Medicine.

[5] Shurson G.C. (2018). Yeast and yeast derivatives in feed additives and ingredients: Sources, characteristics, animal responses, and quantification methods. Anim. Feed Sci. Technol..

[6] Bennett J.W. (1998). Mycotechnology: The role of fungi in biotechnology. J. Biotechnol..

[7] Vohra A., Syal P., Madan A. (2016). Probiotic yeasts in livestock sector. Anim. Feed Sci. Technol..

[8] Ogbuewu I.P., Okoro V.M., Mbajiorgu E.F., Mbajiorgu C.A. (2019). Yeast (*Saccharomyces cerevisiae*) and its effect on production indices of livestock and poultry—A review. Comp. Clin. Pathol..

[9] Bilal R.M., Hassan F.U., Saeed M., Rafeeq M., Zahra N., Fraz A., Saeed S., Khan M.A., Mahgoub H.A., Farag M.R. (2021). Role of Yeast and Yeast-Derived Products as Feed Additives in Broiler Nutrition. Anim. Biotechnol..

[10] Takalloo Z., Nikkhah M., Nemati R., Jalilian N., Sajedi R.H. (2020). Autolysis, plasmolysis and enzymatic hydrolysis of baker’s yeast (*Saccharomyces cerevisiae*): A comparative study. World J. Microbiol. Biotechnol..

[11] Jung E.Y., Hong Y.H., Kim J.H., Park Y., Bae S.H., Chang U.J. (2012). Effects of yeast hydrolysate on hepatic lipid metabolism in high-fat-diet-induced obese mice: Yeast hydrolysate suppresses body fat accumulation by attenuating fatty acid synthesis. Ann. Nutr. Metab..

[12] Bekatorou A., Psarianos C., Koutinas A.A. (2006). Production of food grade yeasts. Food Technol. Biotechnol..

[13] Araujo L.F., Bonato M., Barbalho R., Araujo C.S.S., Zorzetto P.S., Granghelli C.A., Pereira R.J.G., Kawaoku A.J.T. (2018). Evaluating hydrolyzed yeast in the diet of broiler breeder hens. J. Appl. Poult. Res..

[14] Wang T., Cheng K., Yu C.Y., Li Q.M., Tong Y.C., Wang C., Yang Z.B., Wang T. (2021). Effects of a yeast-derived product on growth performance, antioxidant capacity, and immune function of broilers. Poult. Sci..

[15] Sun Z., Wang T., Demelash N., Zheng S., Zhao W., Chen X., Zhen Y., Qin G. (2020). Effect of Yeast Culture (*Saccharomyces cerevisiae*) on Broilers: A Preliminary Study on the Effective Components of Yeast Culture. Animals.

[16] Feye K.M., Carroll J.P., Anderson K.L., Whittaker J.H., Schmidt-McCormack G.R., McIntyre D.R., Pavlidis H.O., Carlson S.A. (2019). *Saccharomyces cerevisiae* fermentation products that mitigate foodborne Salmonella in cattle and poultry. Front. Vet. Sci..

[17] Żółkiewicz J., Marzec A., Ruszczyński M., Feleszko W. (2020). Postbiotics—A step beyond pre-and probiotics. Nutrients.

[18] Kogan G., Kocher A. (2007). Role of yeast cell wall polysaccharides in pig nutrition and health protection. Livest. Sci..

[19] Korolenko T.A., Bgatova N.P., Vetvicka V. (2019). Glucan and mannan—Two peas in a pod. Int. J. Mol. Sci..

[20] Nguyen T.H., Fleet G.H., Rogers P.L. (1998). Composition of the cell walls of several yeast species. Appl. Microbiol. Biotechnol..

[21] Ruiz-Herrera J. (1991). Biosynthesis of beta-glucans in fungi. Antonie Leeuwenhoek Int. J. Gen..

[22] Libjaková L., Bystrický S., Ližičárová I., Paulovičová E., Machová E. (2007). Evaluation of different mannan polysaccharide usage in enzyme-linked immunosorbent assay for specific antibodies determination. J. Pharm. Biomed..

[23] Moreira L.R.S., Filho E.X.F. (2008). An overview of mannan structure and mannan-degrading enzyme systems. Appl. Microbiol. Biotechnol..

[24] Krizková L., Duracková Z., Sandula J., Sasinková V., Krajcovic J. (2001). Antioxidative and antimutagenic activity of yeast cell wall mannans in vitro. Mutat. Res. Genet. Toxicol. Environ. Mutagenesis.

[25] Madrigal-Bujaidar E., Madrigal-Santillán E., Pages N., Kogan G., Chamorro G., Goudey-Perriere F., Bon C., PuisseuxDao S., Sauviat M.-P. (2002). Antigenotoxic studies in mouse to reduce the aflatoxin B1 damage. Toxines et Recherches Biomedicales.

[26] Baumler A.J., Tsolis R.M., Heffron F. (1997). Fimbrial adhesions of Salmonella typhimurium. Role in bacterial interactions with epithelial cells. Adv. Exp. Med. Biol..

[27] Korcová J., Machová E., Filip J., Bystrický S. (2015). Biophysical properties of carboxymethyl derivatives of mannan and dextran. Carbohydr. Polym..

[28] Li J., Karboune S. (2018). A comparative study for the isolation and characterization of mannoproteins from *Saccharomyces cerevisiae* yeast cell wall. Int. J. Biol. Macromol..

[29] Cutler A.J., Botto M., Van Essen D., Rivi R., Davies K.A., Gray D., Walport M.J.T. (1998). Cell dependent immune response in C1q-deficient mice: Defective interferon γ production by antigen-specific T cells. Exp. Med..

[30] Goncharova N.V., Khrapova M.V., Pupyshev A.B., Korolenko E.T., Nešéáková Z., Korolenko T.A. (2016). Hypolipidemic effect of mannan in mice with acute lipemia induced by poloxamer 407. Bull. Exp. Biol. Med..

[31] Jacob J., Pescatore A.J. (2017). Glucans and the Poultry Immune System. Am. J. Immunol..

[32] Zhang B., Guo Y., Wang Z. (2008). The Modulating Effect of β-1, 3/1, 6-glucan Supplementation in the Diet on Performance and Immunological Responses of Broiler Chickens. Asian Australas. J. Anim. Sci..

[33] Omara I.I., Pender C.M., White M.B., Dalloul R.A. (2021). The Modulating Effect of Dietary BetaΒ-Glucan Supplementation on Expression of Immune Response Genes of Broilers during a Coccidiosis Challenge. Animals.

[34] Commins S.P., Borish L., Steinke J.W. (2010). Immunologic messenger molecules: Cytokines, interferons, and chemokines. J. Allergy Clin. Immunol..

[35] Schwartz B., Vetvicka V. (2021). Review: β-glucans as effective antibiotic alternatives in poultry. Molecules.

[36] Morales-Lopez R., Auclair E., Garcia F., Esteve-Garcia E., Brufau J. (2009). Use of yeast cell walls; betaβ-1, 3/1, 6-glucans; and mannoproteins in broiler chicken diets. Poult. Sci..

[37] Šrobárová A., Kogan G., Eged Š. (2005). Yeast polysaccharide affects fusaric acid content in maize root rot. Chem. Biodiv..

[38] Yiannikouris A., François J., Poughon L., Dussap C.G., Bertin G., Jeminet G., Jouany J.P. (2004). Alkali extraction of β-D-glucans from *Saccharomyces cerevisiae* cell wall and study of their adsorptive properties towards Zearalenone. J. Agric. Food Chem..

[39] Yiannikouris A., François J., Poughon L., Dussap C.G., Bertin G., Jeminet G., Jouany J.P. (2004). Adsorption of zearalenone by βD-glucans in the *Saccharomyces cerevisiae* cell wall. J. Food Prot..

[40] Anwar M., Muhammad F., Awais M., Akhtar M. (2017). A review of β-glucans as a growth promoter and antibiotic alternative against enteric pathogens in poultry. World Poult. Sci. J..

[41] Halasz A., Lasztity R. (1991). Use of Yeast Biomass in Food Production.

[42] Jeuniaux C. (1961). Chitinase: An addition to the list of hydrolases in the digestive tract of vertebrates. Nature.

[43] Hossain S.M., Blair R. (2007). Chitin utilisation by broilers and its effect on body composition and blood metabolites. Br. Poult. Sci..

[44] Shahidi F., Abuzaytoun R. (2005). Chitin, chitosan, and co-products: Chemistry, production, applications, and health effects. Adv. Food Nutr..

[45] Jiménez-Gómez C.P., Cecilia J.A. (2020). Chitosan: A Natural Biopolymer with a Wide and Varied Range of Applications. Molecules.

[46] Spring P., Wenk C., Connolly A., Kiers A. (2015). A review of 733 published trials on Bio-Mos^®^, a mannan oligosaccharide, and Actigen®, a second generation mannose rich fraction, on farm and companion animals. E8.; J. Appl. Anim. Nutr..

[47] Yang Y., Iji P.A., Choct M. (2009). Dietary modulation of gut microflora in broiler chickens: A review of the role of six kinds of alternatives to in-feed antibiotics. World Poult. Sci. J..

[48] Xiao R., Power R.F., Mallonee D., Routt K., Spangler L., Pescatore A.J., Cantor A.H., Ao T., Pierce J.L., Dawson K.A. (2012). Effects of yeast cell wall-derived mannan-oligosaccharides on jejunal gene expression in young broiler chickens. Poult. Sci. J..

[49] Tohid T., Hasan G., Alireza T. (2010). Efficacy of mannanoligosaccharides and humate on immune response to Avian Influenza (H9) disease vaccination in broiler chickens. Vet. Res. Commun..

[50] Cohen R.E., Ballou C.E. (1981). Mannoproteins: Structure. Plant carbohydrates II.

[51] Lipke P.N., Ovalle R. (1998). Cell wall architecture in yeast: New structure and new challenges. J. Bacteriol. Res..

[52] Ha C.H., Yun C.W., Paik H.D., Kim S.W., Kang C.W., Hwang H.J., Chang H.I. (2006). Preparation and analysis of yeast cell wall mannoproteins, immune enhancing materials, from cell wall mutant *Saccharomyces cerevisiae*. J. Microbiol. Biotechnol..

[53] Pietrella D., Bistoni G., Corbucci C., Perito S., Vecchiarelli A. (2006). Candida albicans mannoprotein influences the biological function of dendritic cells. Cell. Microbiol..

[54] Johnson C.N., Hashim M.M., Bailey C.A., Byrd J.A., Kogut M.H., Arsenault R.J. (2020). Feeding of yeast cell wall extracts during a necrotic enteritis challenge enhances cell growth, survival and immune signaling in the jejunum of broiler chickens. Poult. Sci. J..

[55] Świątkiewicz S., Arczewska-Włosek A., Józefiak D. (2014). Immunomodulatory efficacy of yeast cell products in poultry: A current review. World Poult. Sci. J..

[56] Gil A. (2002). Modulation of the immune response mediated by dietary nucleotides. Eur. J. Clin. Nutr..

[57] Sauer N., Mosenthin R., Bauer E. (2011). The role of dietary nucleotides in single-stomached animals. Nutr. Res. Rev..

[58] Jung B., Batal A.B. (2012). Effect of dietary nucleotide supplementation on performance and development of the gastrointestinal tract of broilers. Br. Poult. Sci..

[59] Wu C., Yang Z., Song C., Liang C., Li H., Chen W., Lin W., Xie Q. (2018). Effects of dietary yeast nucleotides supplementation on intestinal barrier function, intestinal microbiota, and humoral immunity in specific pathogen-free chickens. Poult. Sci. J..

[60] Feldmann H. (2012). Yeast: Molecular and Cell Biology.

[61] Hill A. (2015). Brewing Microbiology: Managing Microbes, Ensuring Quality and Valorising Waste.

[62] Tofalo R., Suzzi G., Caballero B., Finglas P.M., Toldrá F. (2016). Yeasts. Encyclopedia of Food and Health.

[63] Chemler J.A., Yan Y., Koffas M.A. (2006). Biosynthesis of isoprenoids, polyunsaturated fatty acids and flavonoids in *Saccharomyces cerevisiae*. Microb. Cell Factories.

[64] Pahlavanzadeh M., Sadeghi A.A., Mousavi S.N., Chamani M. (2021). Influence of spleen meal and hydrolyzed yeast on growth performance, blood cells, antibody titres and IL-2 gene expression in broiler chickens, *J*. Appl. Anim. Res..

[65] Sampath V., Han K., Kim I.H. (2021). Influence of yeast hydrolysate supplement on growth performance, nutrient digestibility, microflora, gas emission, blood profile, and meat quality in broilers. J. Anim. Sci. Technol..

[66] Ahiwe E.U., Abdallh M.E., Chang’a E.P., Omede A.A., Al-Qahtani M., Gausi H., Graham H., Ade I.P. (2020). Influence of dietary supplementation of autolyzed whole yeast and yeast cell wall products on broiler chickens. Asian Australas. J. Anim. Sci..

[67] Yalçın S., Eser H., Yalçın S., Cengiz S., Eltan Ö. (2013). Effects of dietary yeast autolysate (*Saccharomyces cerevisiae*) on performance, carcass and gut characteristics, blood profile, and antibody production to sheep red blood cells in broilers. J. Appl. Poult. Res.

[68] Bortoluzzi C., Barbosa J.G.M., Pereira R., Fagundes N.S., Rafael J.M., Menten J.F.M. (2018). Autolyzed Yeast (*Saccharomyces cerevisiae*) Supplementation Improves Performance While Modulating the Intestinal Immune-System and Microbiology of Broiler Chickens. Front. Sustain. Food Syst..

[69] Muthusamy N., Haldar S., Ghosh T.K., Bedford M.R. (2011). Effects of hydrolysed *Saccharomyces cerevisiae* yeast and yeast cell wall components on live performance, intestinal histo-morphology and humoral immune response of broilers. Br. Poult. Sci..

[70] Ahiwe E.U., Abdallh M.E., Chang’a E.P., Al-Qahtani M., Omede A.A., Graham H., Iji P.A. (2019). Influence of autolyzed whole yeast and yeast components on broiler chickens challenged with salmonella lipopolysaccharide. Poult. Sci..

[71] Ullah M., Marri G.M., Jogi Q., Rasheed M., Rizwana H., Kaleri R.R., Goil J.P., Khoso Z.A., Kumar D., Shahab A. (2019). Dietary effect of autolysed yeast on broilers (Levabon^®^, Biomin Austria) on broiler growth. PAB.

[72] Gomez S., Angeles M.D.L. (2011). Effects of an enzymatically hydrolyzed yeast and yeast culture combined with flavomycin and monensin on finishing broiler chickens. Int. J. Poult. Sci.

[73] Bolacali M., Irak K. (2017). Effect of dietary yeast autolysate on performance, slaughter, and carcass characteristics, as well as blood parameters, in quail of both genders. S. Afr. J. Anim. Sci..

[74] Yalçın S., Yalçın S., Cakin K., Eltan O., Dagasan L. (2010). Effects of dietary yeast autolysate (*Saccharomyces cerevisiae*) on performance, egg traits, egg cholesterol content, egg yolk fatty acid composition and humoral immune response of laying hens. J. Sci. Food Agric..

[75] Yalçın S., Yalçın S., Uzunoğlu K., Duyum H.M., Eltan Ö. (2012). Effects of dietary yeast autolysate (*Saccharomyces cerevisiae*) and black cumin seed (*Nigella sativa* L.) on performance, egg traits, some blood characteristics and antibody production of laying hens. Livest. Sci..

[76] Marien M., De Gussem M., Vancraeynest D. Evaluation of a yeast extract product, containing a guaranteed range of β-glucans, in free range laying Hens. Proceedings of the 16th European Symposium on Poultry Nutrition.

[77] Yitbarek A., Rodriguez-Lecompte J.C., Echeverry H.M., Munyaka P., Barjesteh N., Sharif S., Camelo-Jaimes G. (2013). Performance, histomorphology, and Toll-like receptor, chemokine, and cytokine profile locally and ically in broiler chickens fed diets supplemented with yeast-derived macromolecules. Poult. Sci..

[78] Attia Y.A., Al-Khalaifah H., Ibrahim M.S., Abd Al-Hamid A.E., Al-Harthi M.A., El-Naggar A. (2017). Blood Hematological and Biochemical Constituents, Antioxidant Enzymes, Immunity and Lymphoid Organs of Broiler Chicks Supplemented with Propolis, Bee Pollen and Mannan Oligosaccharides Continuously or Intermittently. Poult. Sci..

[79] Ahiwe E.U., Chang’a E.P., Abdallh M.E., Al-Qahtani M., Kheravii S.K., Wu S., Graham H., Iji P.A. (2019). Dietary hydrolysed yeast cell wall extract is comparable to antibiotics in the control of subclinical necrotic enteritis in broiler chickens. Br. Poult. Sci..

[80] Chou W.K., Park J., Carey J.B., McIntyre D.R., Berghman L.R. (2017). Immunomodulatory effects of *Saccharomyces cerevisiae* fermentation product supplementation on immune gene expression and lymphocyte distribution in immune organs in broilers. Front. Vet. Sci..

[81] Gao J., Zhang H.J., Yu S.H., Wu S.G., Yoon I., Quigley J., Gao Y.P., Qi G.H. (2008). Effects of yeast culture in broiler diets on performance and immunomodulatory functions. Poult. Sci..

[82] Gao J., Zhang H.J., Wu S.G., Yu S.H., Yoon I., Moore D., Gao Y.P., Yan H.J., Qi G.H. (2009). Effect of *Saccharomyces cerevisiae* fermentation product on immune functions of broilers challenged with Eimeria tenella. Poult. Sci..

[83] Liu Y., Cheng X., Zhen W., Zeng D., Qu L., Wang Z., Ning Z. (2021). Yeast culture improves egg quality and reproductive performance of aged breeder layers by regulating gut microbes. Front. Microbiol..

[84] Chuang W.Y., Lin L.J., Hsieh Y.C., Chang S.C., Lee T.T. (2021). Effects of *Saccharomyces cerevisiae* and phytase co-fermentation of wheat bran on growth, antioxidation, immunity and intestinal morphology in broilers. Anim. Biosci..

[85] Chuang W.Y., Lin W.C., Hsieh Y.C., Huang C.M., Chang S.C., Lee T. (2019). TEvaluation of the combined use of Saccharomyces cerevisiae and Aspergillus oryzae with phytase fermentation products on growth, inflammatory, and intestinal morphology in broilers. Animals.

[86] Nidamanuri A.L., Leslie Leo Prince L., Yadav S.P., Bhattacharya T.K., Konadaka S.R.R., Bhanja S.K. (2021). Effect of Supplementation of Fermented Yeast Culture on Hormones and Their Receptors on Exposure to Higher Temperature and on Production Performance after Exposure in Nicobari Chickens. Int. J. Endocrinol..

[87] Nidamanuri A.L., Prince L.L.L., Mahapatra R.K., Murugesan S. (2022). Effect on physiological and production parameters upon supplementation of fermented yeast culture to Nicobari chickens during and post summer. J. Anim. Physiol. Anim. Nutr..

[88] Laxmi N.A., Ramasubbaiah K., Mahapatra R.K., Shanmugam M. (2019). Effect of Supplementation of Fermented Yeast Culture During Summer on Plasma Leptin and Ghrelin and Expression of their Receptors in Different Tissues and on Production Performance During-Post Summer Period in PD 3 Chicken Line. Anim. Nutr. Feed Technol..

[89] Nidamanuri A.L., Lawerence L.L.P., Kothamidde R.S., Mahapatra R.K. (2019). Relationship Between Plasma GH, Metabolites, Lipogenic Genes, and MMP3 Expression in PD3 Chicken Line and Role of Fermented Yeast Culture in Alleviating Heat Stress. J. Appl. Poult. Res..

[90] Park J.H., Sureshkumar S., Kim I.H. (2020). Egg production, egg quality, nutrient digestibility, and excreta microflora of laying hens fed with a diet containing brewer’s yeast hydrolysate. J. Appl. Anim. Res..

[91] Sun Z., Wang T., Aschalew N.D., Zhao W., Chen X., Zhang X.F., Zhen Y.G., Qin G.X. (2020). Effects of yeast cultures with different fermentation times on the growth performance, caecal microbial community and metabolite profile of broilers. J. Anim. Physiol. Anim. Nutr..

[92] Feye K.M., Rubinelli P.M., Chaney W.E., Pavlidis H.O., Kogut M.H., Ricke S.C. (2020). The preliminary development of an in vitro poultry cecal culture model to evaluate the effects of Original XPCTM for the reduction of Campylobacter jejuni and its potential effects on the microbiota. Front. Microbiol..

[93] Zhao W., Zhen Y.G., Zhang X.F., Li L.J., Chen X., Wang T., Lee H.G. (2019). Effects of yeast culture on broiler growth performance, nutrient digestibility and caecal microbiota. S. Afr. J. Anim. Sci..

[94] Feye K.M., Dittoe D.K., Rubinelli P.M., Olson E.G., Ricke S.C. (2021). Yeast Fermentate-Mediated Reduction of Salmonella Reading and Typhimurium in an in vitro Turkey Cecal Culture Model. Front. Microbiol..

[95] Roto S.M., Park S.H., Lee S.I., Kaldhone P., Pavlidis H.O., Frankenbach S.B., McIntyre D.R., Striplin K., Brammer L., Ricke S.C. (2017). Effects of feeding Original XPC™ to broilers with a live coccidiosis-vaccine under industry conditions: Part 1. Growth performance and Salmonella inhibition. Poult. Sci..

[96] Rubinelli P., Roto S., Kim S., Park S.H., Pavlidis H.O., McIntyre D., Ricke S.C. (2016). Reduction of Salmonella Typhimurium by fermentation metabolites of Diamond V Original XPC in an in vitro anaerobic mixed chicken cecal culture. Front. Vet. Sci..

[97] Gingerich E., Frana T., Logue C.M., Smith D.P., Pavlidis H.O., Chaney W.E. (2021). Effect of feeding a postbiotic derived from *Saccharomyces Cerevisiae* fermentation as a preharvest food safety hurdle for reducing Salmonella Enteritidis in the ceca of layer pullets. J. Food Prot..

[98] Soleimanpour Rakhneh A., Khalaji S., Yari M., Ghabooli M. (2020). Evaluating the efficacy of plant-specific fungus (Piriformospora indica) rich in mannan oligosaccharides as a microbial feed additive on growth performance, protein digestibility, plasma characteristics, intestinal microflora, and morphology in chicks. J. Appl. Anim. Res..

[99] Akhavan-Salamat H., Ghasemi H.A., Khaltabadi-Farahani A.H., Kazemi-Bonchenari M. (2011). The effects of *Saccharomyces cerevisiae* on performance and nutrients digestibility in broilers fed with diet containing different levels of phosphorous. Afr. J. Biotechnol..

[100] Zhang J.C., Chen P., Zhang C., Khalil M.M., Zhang N.Y., Qi D.S., Wang Y.W., Sun L.H. (2020). Yeast culture promotes the production of aged laying hens by improving intestinal digestive enzyme activities and the intestinal health status. Poult. Sci..

[101] Zhang A.W., Lee B.D., Lee S.K. (2005). Effects of yeast (*Saccharomyces cerevisiae*) cell components on growth performance, meat quality, and ileal mucosa development of broiler chicks. Poult. Sci..

[102] Panda A.K., Rama Rao S.V., Raju M.V.L.N., Sharma S.R. (2006). Dietary supplementacion of lactobacillus sporogenes on performance and serum biochemico-lipid profile of broiler chickens. J. Poult. Sci..

[103] Sacakli P., Ergun A., Koksal B.H., Bayraktaroglu A.G., Sizmaz O. (2011). Effects of diets supplemented with yeast (*Saccharomyces cerevisiae*) products or/and hops (*Humulus iupulus*) on growth perfor-mance and intestinal morphology in broilers. Revue Méd. Vét..

[104] Fathi M.M., Al-Mansour S., Al-Homidan A., Al-Khalaf A., Al-Damegh M. (2012). Effect of yeast culture supplementation on carcass yield and humoral immune response of broiler chicks. Vet. World.

[105] Hoque M.R., Jung H.I., Kim I.H. (2020). Effect of Yeast Culture (*Saccharomyces cerevisiae*) Supplementation on Growth Performance, Excreta Microbes, Noxious Gas, Nutrient Utilization, and Meat Quality of Broiler Chicken. J. Poult. Sci..

[106] Kiros T.G., Gaydos T., Corley J., Raspoet R., Berghaus R., Hofacre C. (2019). Effect of *Saccharomyces cerevisiae* yeast products in reducing direct colonization and horizontal transmission of Salmonella Heidelberg in broilers. J. Appl. Poult. Res..

[107] Al-Mansour S., Al-Khalf A., Al-Homidan I., Fathi M.M. (2011). Feed efficiency and blood hematology of broiler chicks given a diet supplemented with yeast culture. Int. J. Poult. Sci..

[108] Feye K.M., Anderson K.L., Scott M.F., McIntyre D.R., Carlson S.A. (2016). Inhibition of the virulence, antibiotic resistance, and fecal shedding of multiple antibiotic-resistant Salmonella Typhimurium in broilers fed Original XPC™. Poult. Sci..

[109] Osweiler G.D., Jagannatha S., Trampel D.W., Imerman P.M., Ensley S.M., Yoon I., Moore D.T. (2010). Evaluation of XPC and prototypes on aflatoxin-challenged broilers. Poult. Sci..

[110] Sobotik E.B., Nelson J.R., Pavlidis H.O., Archer G.S. (2022). Evaluating the effects of supplementing *Saccharomyces cerevisiae* in the feed or drinking water on stress susceptibility of broilers. J. Appl. Anim. Res..

[111] Elliott K.E.C., Branton S.L., Evans J.D., Leigh S.A., Kim E.J., Olanrewaju H.A., Pharr G.T., Pavlidis H.O., Gerard P.D., Peebles E.D. (2020). Growth and humoral immune effects of dietary Original XPC in layer pullets challenged with Mycoplasma gallisepticum. Poult. Sci..

[112] Yalçin S., Özsoy B., Erol H. (2008). Yeast culture supplementation to laying hen diets containing soybean meal or sunflower seed meal and its effect on performance, egg quality traits, and blood chemistry. J. Appl. Anim. Res..

[113] Bartz B.M., McIntyre D.R., Grimes J.L. (2018). Effects of Management Related Practices on Turkey Hen Performance Supplemented with Either Original XPC™ or AviCare™. Front. Vet. Sci..

[114] Martinez J.S., Blount R.L., Park J., McIntyre D.R., Pavlidis H.O., Carey J.B. (2018). Effects of feeding original XPCTM to laying hens on egg production, component yield and composition. J. Appl. Anim. Res..

[115] Özsoy B., Karadağoğlu Ö., Yakan A., Önk K., Çelik E., Şahin T. (2018). The role of yeast culture (*Saccharomyces cerevisiae*) on performance, egg yolk fatty acid composition, and fecal microflora of laying hens. Rev. Bras. Zootec..

[116] Lensing M., Van der Klis J.D., Yoon I., Moore D.T. (2012). Efficacy of *Saccharomyces cerevisiae* fermentation product on intestinal health and productivity of coccidian-challenged laying hens. Poult. Sci..

[117] Özsoy B., Yalçın S. (2011). The effects of dietary supplementation of yeast culture on performance, blood parameters and immune system in broiler turkeys. Ankara Üniv. Vet. Fak. Derg..

[118] Bradley G.L., Savage T.F. (1995). The effect of autoclaving a yeast culture of *Saccharomyces cerevisiae* on turkey poult performance and the retention of gross energy, and selected minerals. Anim. Feed Sci. Technol..

[119] Čepulienė R., Gudavičiūtė D., Semaška V., Bobinienė R., Kepalienė I., Vencius D., Sirvydis V. (2010). Effect of the yeast culture feed additive on productivity and egg quality of laying quails. Vet. Zootech..

[120] Abdelrahman M.M. (2013). Effects of feeding dry fat and yeast culture on broiler chicken performance. Turk. J. Vet. Anim. Sci..

[121] Firman J.D., Moore D., Broomhead J., McIntyre D. (2013). Effects of dietary inclusion of a *Saccharomyces cerevisiae* fermentation product on performance and gut characteristics of male turkeys to market weight. Int. J. Poult. Sci..

[122] Aristides L.G.A., Venancio E.J., Alfieri A.A., Otonel R.A.A., Frank W.J., Oba A. (2018). Carcass characteristics and meat quality of broilers fed with different levels of *Saccharomyces cerevisiae* fermentation product. Poult. Sci..

[123] Nelson J.R., Sobotik E.B., Athrey G., Archer G.S. (2020). Effects of supplementing yeast fermentate in the feed or drinking water on stress susceptibility, plasma chemistry, cytokine levels, antioxidant status, and stress-and immune-related gene expression of broiler chickens. Poult. Sci..

[124] Wenner C.A., Güler M.L., Macatonia S.E., O’Garra A., Murphy K.M. (1996). Roles of IFN-gamma and IFN-alpha in IL-12-induced T helper cell-1 development. J. Immunol..

[125] Le Bon A., Durand V., Kamphuis E., Thompson C., Bulfone-Paus S., Rossmann C., Tough D.F. (2006). Direct stimulation of T cells by type I IFN enhances the CD8+ T cell response during cross-priming. J. Immunol..

[126] Nelson J.R., Ibrahim M.M., Sobotik E.B., Athrey G., Archer G.S. (2020). Effects of yeast fermentate supplementation on cecal microbiome, plasma biochemistry and ileal histomorphology in stressed broiler chickens. Livest. Sci..

[127] Nelson J.R., Archer G.S. (2019). Effect of yeast fermentate supplementation on intestinal health and plasma biochemistry in heat-stressed Pekin ducks. Animals.

[128] Jensen G.S., Patterson K.M., Yoon I. (2008). Yeast culture has anti-inflammatory effects and specifically activates NK cells. Comp. Immunol. Microbiol. Infect. Dis..

[129] Stier H., Ebbeskotte V., Gruenwald J. (2014). Immune-modulatory effects of dietary Yeast Beta-1, 3/1, 6-D-glucan. Nutr. J..

[130] Chacher M.F.A., Kamran Z., Ahsan U., Ahmad S., Koutoulis K.C., Din H.Q.U., Cengiz Ö. (2017). Use of mannan oligosaccharide in broiler diets: An overview of underlying mechanisms. Worlds Poult. Sci. J..

[131] Torok V.A., Hughes R.J., Ophel-Keller K., Ali M., MacAlpine R. (2009). Influence of different litter materials on cecal microbiota colonization in broiler chickens. Poult. Sci..

[132] Lozupone C.A., Stombaugh J.I., Gordon J.I., Jansson J.K., Knight R. (2012). Diversity, stability and resilience of the human gut microbiota. Nature.

[133] Rinttilä T., Apajalahti J. (2013). Intestinal microbiota and metabolites—Implications for broiler chicken health and performance. J. Appl. Poult. Res..

[134] Park S.H., Roto S., Pavlidis H., McIntyre D., Striplin K., Brammer L., Ricke S.C. (2017). Effects of feeding Original XPC™ to broilers with a live coccidiosis vaccine under industrial conditions: Part 2. Cecal microbiota analysis. Poult. Sci..

[135] Park S.H., Kim S., Lee S.I., Rubinelli P.M., Roto S.M., Pavlidis H.O., McIntyre D., Ricke S.C. (2017). Original XPCTM effect on Salmonella Typhimurium and cecal microbiota from three different ages of broiler chickens when incubated in an anaerobic in vitro culture system. Front. Microbiol..

[136] Zafar H., Saier Jr M.H. (2021). Gut Bacteroides species in health and disease. Gut Microbes.

[137] Eeckhaut V., Van Immerseel F., Croubels S., De Baere S., Haesebrouck F., Ducatelle R., Louis P., Vandamme P. (2011). Butyrate production in phylogenetically diverse Firmicutes isolated from the chicken caecum. Microb. Biotechnol..

[138] Mishra B., Jha R. (2019). Oxidative stress in the poultry gut: Potential challenges and interventions. Front. Vet. Sci..

[139] Ahiwe E.U., Dos Santos T.T., Graham H., Iji P.A. (2021). Can probiotic or prebiotic yeast (*Saccharomyces cerevisiae*) serve as alternatives to in-feed antibiotics for healthy or disease-challenged broiler chickens—A review. J. Appl. Poult. Res..

